# The Neuron-Astrocyte-Microglia Triad in Normal Brain Ageing and in a Model of Neuroinflammation in the Rat Hippocampus

**DOI:** 10.1371/journal.pone.0045250

**Published:** 2012-09-18

**Authors:** Francesca Cerbai, Daniele Lana, Daniele Nosi, Polina Petkova-Kirova, Sandra Zecchi, Holly M. Brothers, Gary L. Wenk, Maria Grazia Giovannini

**Affiliations:** 1 Department of Pharmacology, University of Florence, Florence, Italy; 2 Department of Anatomy, Histology and Legal Medicine, University of Florence, Florence, Italy; 3 Institute of Biophysics, Bulgarian Academy of Sciences, Sofia, Bulgaria; 4 Department of Psychology, The Ohio State University, Columbus, Ohio, United States of America; Univ. Kentucky, United States of America

## Abstract

Ageing is accompanied by a decline in cognitive functions; along with a variety of neurobiological changes. The association between inflammation and ageing is based on complex molecular and cellular changes that we are only just beginning to understand. The hippocampus is one of the structures more closely related to electrophysiological, structural and morphological changes during ageing. In the present study we examined the effect of normal ageing and LPS-induced inflammation on astroglia-neuron interaction in the rat hippocampus of adult, normal aged and LPS-treated adult rats. Astrocytes were smaller, with thicker and shorter branches and less numerous in CA1 Str. radiatum of aged rats in comparison to adult and LPS-treated rats. Astrocyte branches infiltrated apoptotic neurons of aged and LPS-treated rats. Cellular debris, which were more numerous in CA1 of aged and LPS-treated rats, could be found apposed to astrocytes processes and were phagocytated by reactive microglia. Reactive microglia were present in the CA1 Str. Radiatum, often in association with apoptotic cells. Significant differences were found in the fraction of reactive microglia which was 40% of total in adult, 33% in aged and 50% in LPS-treated rats. Fractalkine (CX3CL1) increased significantly in hippocampus homogenates of aged and LPS-treated rats. The number of CA1 neurons decreased in aged rats. In the hippocampus of aged and LPS-treated rats astrocytes and microglia may help clearing apoptotic cellular debris possibly through CX3CL1 signalling. Our results indicate that astrocytes and microglia in the hippocampus of aged and LPS-infused rats possibly participate in the clearance of cellular debris associated with programmed cell death. The actions of astrocytes may represent either protective mechanisms to control inflammatory processes and the spread of further cellular damage to neighboring tissue, or they may contribute to neuronal damage in pathological conditions.

## Introduction

Until recently, neurons were considered to be the basic functional units of the central nervous system, while glia cells only to serve as supportive elements. This concept is rapidly changing; it is becoming more evident that proper functioning of the neuron-microglia-astrocyte “triad” is fundamental for the functional organization of the brain [1], [2]. Impaired interplay among neurons and glia may be responsible for derangements from normal brain aging to neurodegenerative processes [3], [4]. Recruitment and activation of glial cells in a complex temporal pattern require well organized reciprocal communication between neurons and glia as well as among glial cells. Therefore, it is critical to better understand the interactions among neurons, astrocytes and microglia, the so-called “triad”, in physiological and during pathological processes. In the current study we used two animal models that reproduce vital components that predispose the brain to degeneration: aging and chronic neuroinflammation. The interactions between neurons, astrocytes and microglia were compared in normal aged rats and adult rats infused with lipopolysaccharide (LPS) into the 4th ventricle. We have extensively characterized the slowly evolving and region specific series of changes induced by the chronic infusion of picomolar levels of LPS; these changes likely represent a complex interplay of glial and neuronal interactions leading to a series of compensatory changes that occur following the development of tolerance to the presence of LPS. We have previously demonstrated [5] that after two days of LPS infusion activated microglia were seen diffusely scattered throughout the brain, suggesting that the LPS had spread throughout both cerebral hemispheres within a relatively short period of time. During the next two weeks, the number of activated microglia gradually decreased in all regions with some key exceptions; following four weeks of LPS infusion the greatest inflammatory response was concentrated within the hippocampus [5]. After four weeks of LPS infusion, MRI studies identified enlarged lateral ventricles with shrinkage of the temporal lobe regions similar to that observed in patients during the initial stages of mild cognitive impairment [6]. Taken together, these findings suggest that LPS quickly initiates a cascade of biochemical processes that show time-dependent, regional and cell specific changes that are maximal after four weeks of LPS infusion [5], [7]. Recently, Franceschi and coworkers [8] introduced the term "inflammaging" which describes the progressive changes which occur in the ageing brain, characterized by a low-grade chronic up-regulation of certain pro-inflammatory responses. Thus, we studied the modifications of neuroinflammatory markers present in the brain of aged rats as well as in a more aggressive form of brain inflammation such as that induced by injection of LPS in the fourth ventricle of adult rats to verify whether a form of neuroinflammaging might also be present; a comparison between the two models was also performed.

How apoptosis causes neurons to die is still a matter of debate,however the principle mechanism is by triggering the release of intercellular signals which induce phagocytic cells to consume the neuron [9]. Astrocytes and microglia express membrane receptors that recognize molecules released by neurons [9], [10], leading to the phagocytosis of 'altered' cells and neuronal debris.

The current study focused on changes in these intercellular signals in the hippocampus because of its critical role in memory processing [11] and because it demonstrates significant functional, structural, and morphological alterations with ageing and dementia. We investigated how the interaction between glia cells and neurons change during normal ageing or in response to LPS-induced chronic neuroinflammation in comparison to normal adult rats. We describe how astrocytes and microglia in the hippocampus of aged and LPS-infused adult rats participate in the clearance of cellular debris associated with programmed cell death.

## Materials and Methods

### Animals

Male Wistar rats, 3 (adult) and 22 months old (aged, Harlan Nossan, Milano, Italy), were used. The animals were individually housed in macrolon cages with ad libitum food and water and were maintained on a 12 h light–12 h dark cycle with light at 7∶00 am in a temperature-controlled room (23±1°C). Experiments on aged and normal adult rats were performed in the Department of Pharmacology, University of Florence, Italy and animal manipulations on aged rats and retalive controls were carried out according to the Italian Guidelines for Animal Care (DL 116/92, application of the European Communities Council Directive 86/609/EEC) and approved by the local IACUC. Experiments on LPS- and acsf- treated rats were performed in the Department of Psychology, The Ohio State University, Columbus, OH 43210, USA in accordance with the National Institute of Health Guide for the Care and Use of Laboratory Animals (NIH Publications No. 80–23) revised 1996; formal approval to conduct the experiments was obtained from the animal subjects review board from Ohio State University (Institutional Animal Care and Use Committee; approval number 2008A0028). All efforts were made to minimize animal sufferings and to use only the number of animals necessary to produce reliable scientific data.

**Figure 1:**
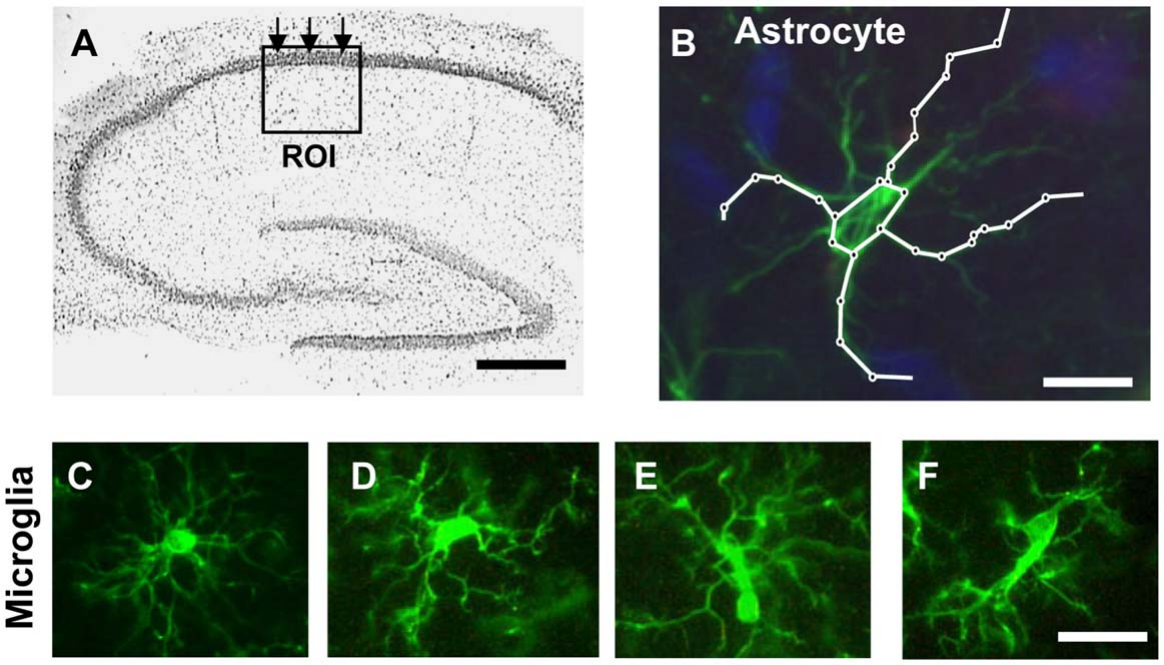
Methods: ROI, scheme of measure of astrocytes branches lenght, examples of resting-reactive microglia. **A:** localization and dimensions of the Region Of Interest (ROI) utilized to perform the quantitative analysis; scale bar: 500 µm. **B:** schematic diagram showing the method used to quantify principal astrocytes branches length; scale bar: 15 µm. **C–F:** different stages of microglia activation. **C:** a typical example of a resting microglia cell; **D–F**: typical examples of microglia in reactive states; scale bar: 20 µm.

### Surgery

LPS or artificial cerebrospinal fluid (aCSF) was chronically infused for 4 weeks through a cannula, implanted into the 4th ventricle of adult rats, attached to an osmotic minipump as previously described [7]. Body weight was determined daily and general behavior was monitored for normal grooming behavior and seizures. The Alzet osmotic minipump containing LPS (Sigma; *E. coli*, serotype 055:B5, TCA extraction; 1.6 µg/ml) was implanted into the dorsal abdomen of the rat and attached via Tygon tubing (0.060" O.D.) to a chronic indwelling cannula (Model 3280P, osmotic pump connect, 28 gauge, Plastics One, Inc., Roanoke, VA) that was positioned stereotaxically so that the cannula tip extended to these coordinates within the 4th ventricle: 2.5 mm posterior to Lambda, on the midline, and 7 mm ventral to the dura. Controls were infused with aCSF: (in mM) 140 NaCl; 3.0 KCl; 2.5 CaCl_2_; 1.0 MgCl_2_; 1.2 Na_2_HPO_4_, pH 7.4. The osmolarity of the solution within the minipumps was adjusted when the LPS was added so that it was isosmolar with the brain (i.e., 320 mOs) by decreasing the amount of added glucose within the aCSF. A volume overload to the brain is minimal using this procedure because the 0.15 ul/hr administered contributes only about 0.16% of the total CSF volume produced by the rat each hour and is only 0.09% of the rat’s total CSF volume. Post-operative care included chloramphenicol (1% solution) applied to the exposed skull and scalp prior to closure, Bupivicaine (a topical anesthetic) applied locally to the scalp to lessen pain, and 4 ml of sterile isotonic saline injected (s.c.) to prevent dehydration during recovery. Isoflurane gas was used to induce anesthesia during the implantation of the osmotic minipumps. LPS-infused rats were sacrificed precisely four weeks after the initiation of the infusion. For any further detail refer to [7], [12].

**Figure 2:**
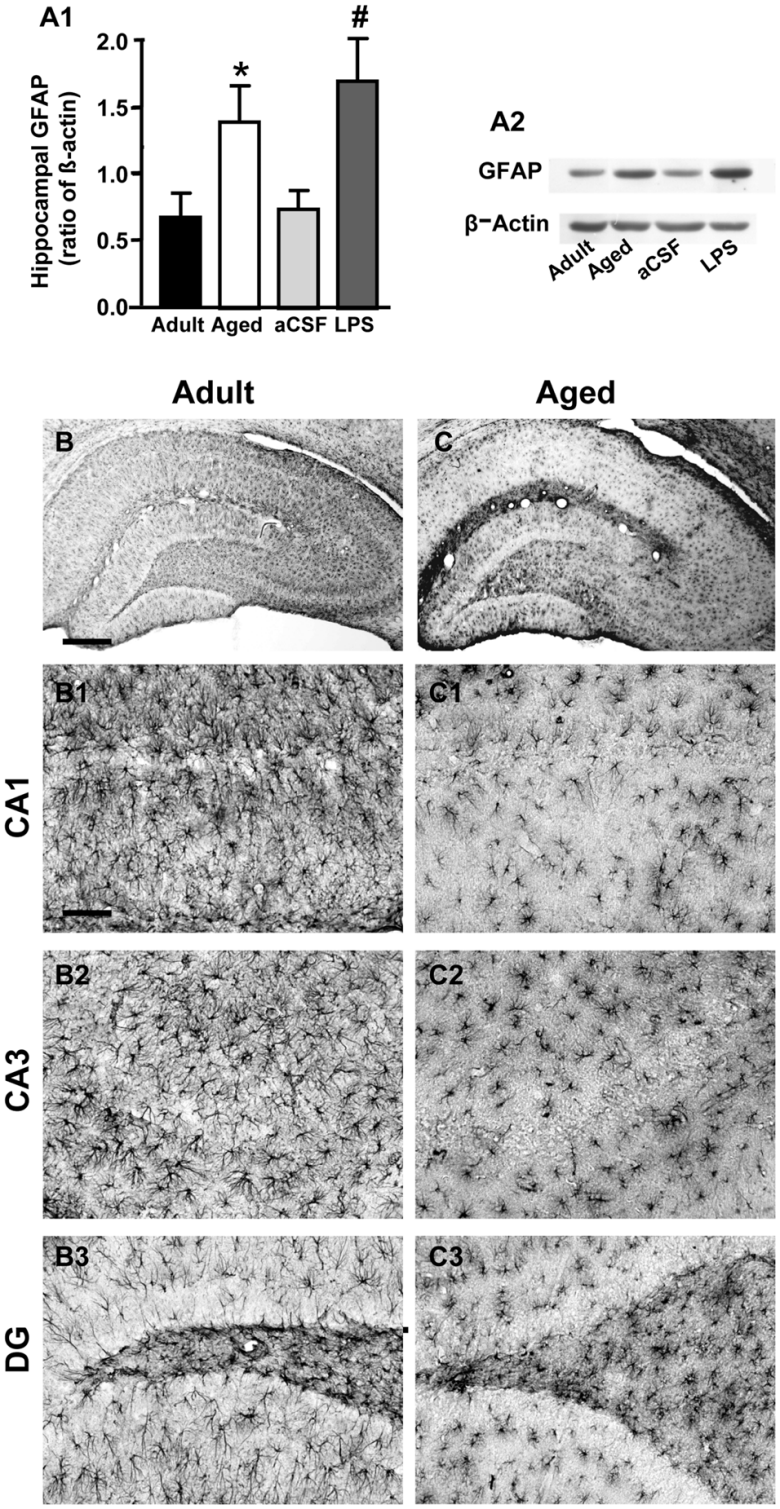
Western Blot analysis of GFAP levels in hippocampus and immunohistochemistry of GFAP positive cells. A1: quantification of GFAP by Western Blot from homogenates of whole hippocampus. Each column represents the levels of GFAP expressed as a ratio of β-actin expression run in the same gel (mean ± SEM; Adult, n = 8; aged, n = 5; aCSF, n = 6; LPS-treated, n = 6) **A2:** representative Western Blot runs of GFAP and β-actin. **B,C**: immunolabelling of astrocytes using anti GFAP antibody and DAB staining in whole hippocampal slices. **B1–B3**: higher magnification images of CA1 (**B1**), CA3 (**B2**) and DG (**B3**); **C1–C3**: higher magnification images of CA1 (**C1**), CA3 (**C2**) and DG (**C3**); **B-B3**: adult rat; **C–C3**: aged rat. Scale bar: **B–C**: 400 µm; **B1–C3**∶70 µm.

### Immunohistochemistry

Rats were anesthetized with chloral hydrate (400 g/kg) and perfused transcardially with 500 ml of ice-cold paraformaldehyde (4% in phosphate-buffered saline, PBS, pH 7.4). The brains were postfixed for 4 h and cryoprotected in 18% sucrose/PBS solution for at least 48 h. Coronal sections (40 µm) were cut with a cryostat, placed in 1 ml of anti-freeze solution and stored at −20°C until immunohistochemistry.

**Figure 3:**
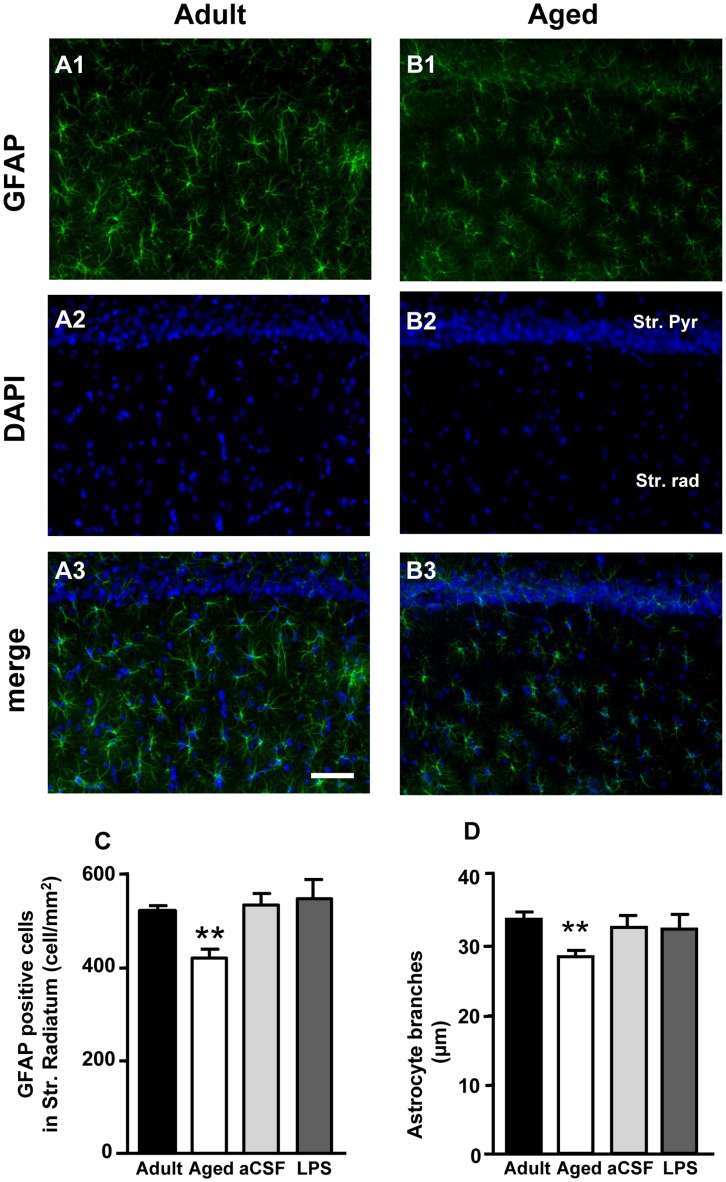
Quantitative analysis of astrocytes in CA1 Str. Radiatum. Characterization of astrocytes in CA1 Str. Radiatum of adult and aged rats. Representative epifluorescent photomicrographs showing immunoreactivity of GFAP (green) and DAPI staining (blue) in CA1 Pyramidal cell layer and Str. Radiatum of adult (**A1,A2,A3**) and aged (**B1,B2,B3**) rats. **A3** and **B3** show the merged images. Scale bar: 50 µm. **C:** quantitative analysis of GFAP positive cells counted in CA1 Str. Radiatum of adult (n = 12), aged (n = 15), aCSF- (n = 5) and LPS-treated (n = 6) rats, expressed as GFAP positive cells/mm^2^ (mean±SEM); **P<0.01 vs all other groups. **D:** length of principal astrocyte branches in CA1 Str. Radiatum of adult (n = 12), aged (n = 15), aCSF- (n = 5) and LPS-treated (n = 6) rats; (mean±SEM), **P<0.01 vs all other groups.

#### Antibodies

The following primary antibodies were used: a mouse monoclonal anti-neuronal nuclei (NeuN, 1∶200; Chemicon, Temecula, CA, USA) for neurons; a rabbit polyclonal anti-glial fibrillary acidic protein (GFAP, 1∶1000; DakoCytomation, Glostrup, Denmark) for astrocytes; a rabbit polyclonal IBA1 (1∶300, WAKO Pure Chem. Ind, Osaka, Japan) for microglia. When triple staining for astrocytes, microglia and neurons was performed, to prevent possible cross-reactions between primary and secondary antibodies, we visualized astrocytes with a mouse monoclonal anti-GFAP antibody directly conjugated with the fluorochrome AlexaFluor 488 (1∶500; Millipore, Billerica, MA). Rabbit polyclonal anti-phospho-(Thr180/Tyr182)-p38MAPK antibody (1∶250) or anti-phospho-(Thr183/Tyr185)-JNK antibody (1∶1000) were used for the activated forms of p38MAPK and JNK, respectively (all from Cell Signaling Technology, Inc., Danvers, MA, USA). Each antibody specifically recognizes the phosphorylated form of the respective kinase, with no cross-reaction to the other one. Apoptosis was examined using two different antibodies, one against Cytochrome C (mouse monoclonal, 1∶200, Becton and Dickinson, Franklin Lakes, NJ, USA) and the other against the Apoptosis Inducing Factor (AIF, goat polyclonal 1∶100, Santa Cruz Biotechnology, Santa Cruz, CA, USA). Connexin43 (Cx43) was visualized with a rabbit polyclonal antibody (1∶50, Santa Cruz Biotechnology) and CX3CL1 with a rabbit polyclonal antibody (1∶400, Abcam, Cambridge, UK).

**Figure 4:**
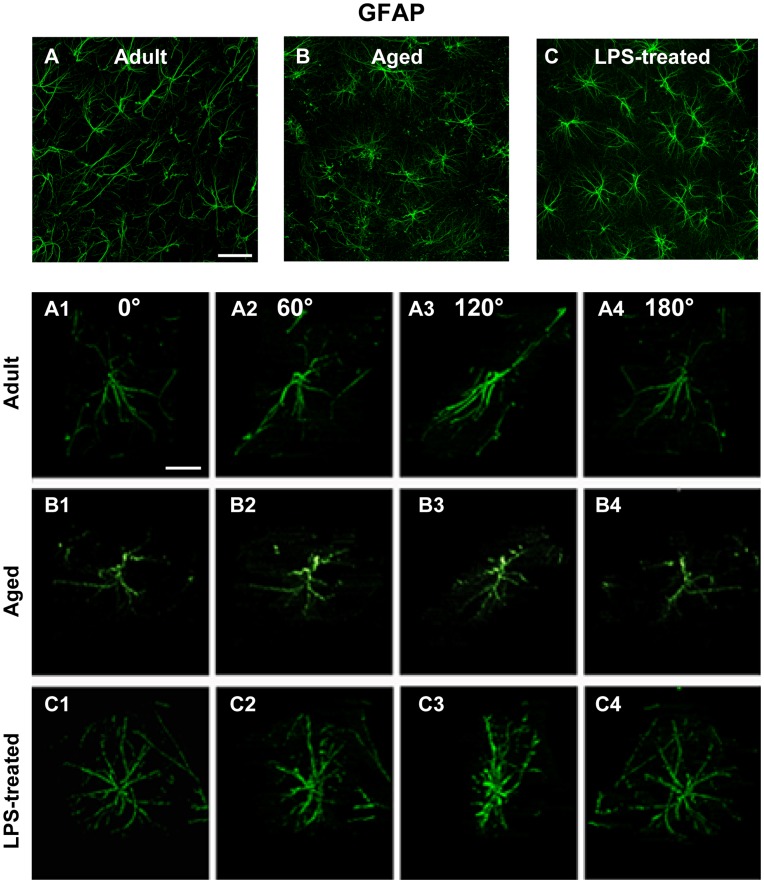
Confocal microscopy 3D-analysis of astrocytes morphology. Immunoreactivity of GFAP in the CA1 Str. Radiatum of adult (**A, A1–A4**), aged (**B, B1–B4**) and LPS-treated rats (**C, C1–C4**). Panels from **A1** to **C4** are obtained from the 3D stacks observed from different angles (0, 60, 120, 180 degrees) around the vertical axis. Scale bar: 40 µm (**A,B,C**) and 15 µm (**A1–C4**).

**Figure 5:**
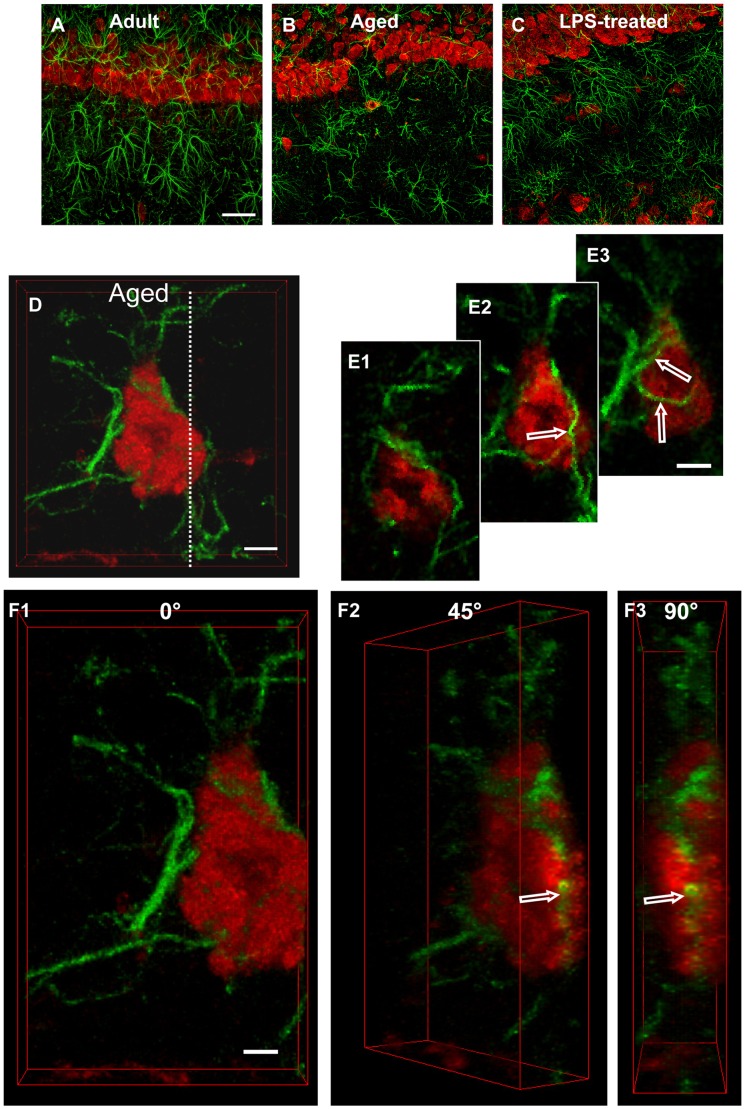
Characterization of astrocytes-neurons interplay. Photos show confocal images of immunoreactivity of GFAP (green) and NeuN (red) in CA1 Pyramidal cell layer and CA1 Str. Radiatum of adult (**A**), aged (**B** and **D, E1–F3**) and LPS-treated rats (**C**). Scale bar: 60 µm (**A**,**B**,**C**). **D:** 3D stack of confocal scans of GFAP (green), and NeuN (red). Scale bar: 5 µm. **E1–E3:** each panel is obtained merging 2 consecutive confocal scans (total 0.738 µm). Scale bar: 5 µm. **F1–F3∶**3D stacks of the neuron shown in D, digitally cut along the white dotted line and rotated by 0, 45 and 90 degrees along the vertical axis. Scale bar: 3 µm.

The following secondary antibodies were used: for DAB staining we used biotinylated goat anti-rabbit, diluted 1∶333 or anti-mouse diluted 1∶1000, as necessary (both from Vectastain, Vector Laboratories). Fluorescent secondary antibodies: AlexaFluor 488 donkey anti-rabbit (1∶400), AlexaFluor 594 goat anti-mouse (1∶400), AlexaFluor 633 donkey anti-goat (1∶400), AlexaFluor 555 donkey anti-mouse (1∶400), AlexaFluor 594 donkey anti-goat (1∶400), Alexa Fluor 635 goat anti-rabbit (1∶400) as necessary (all from InVitrogen Co, Carlsbad, CA, USA).

**Figure 6:**
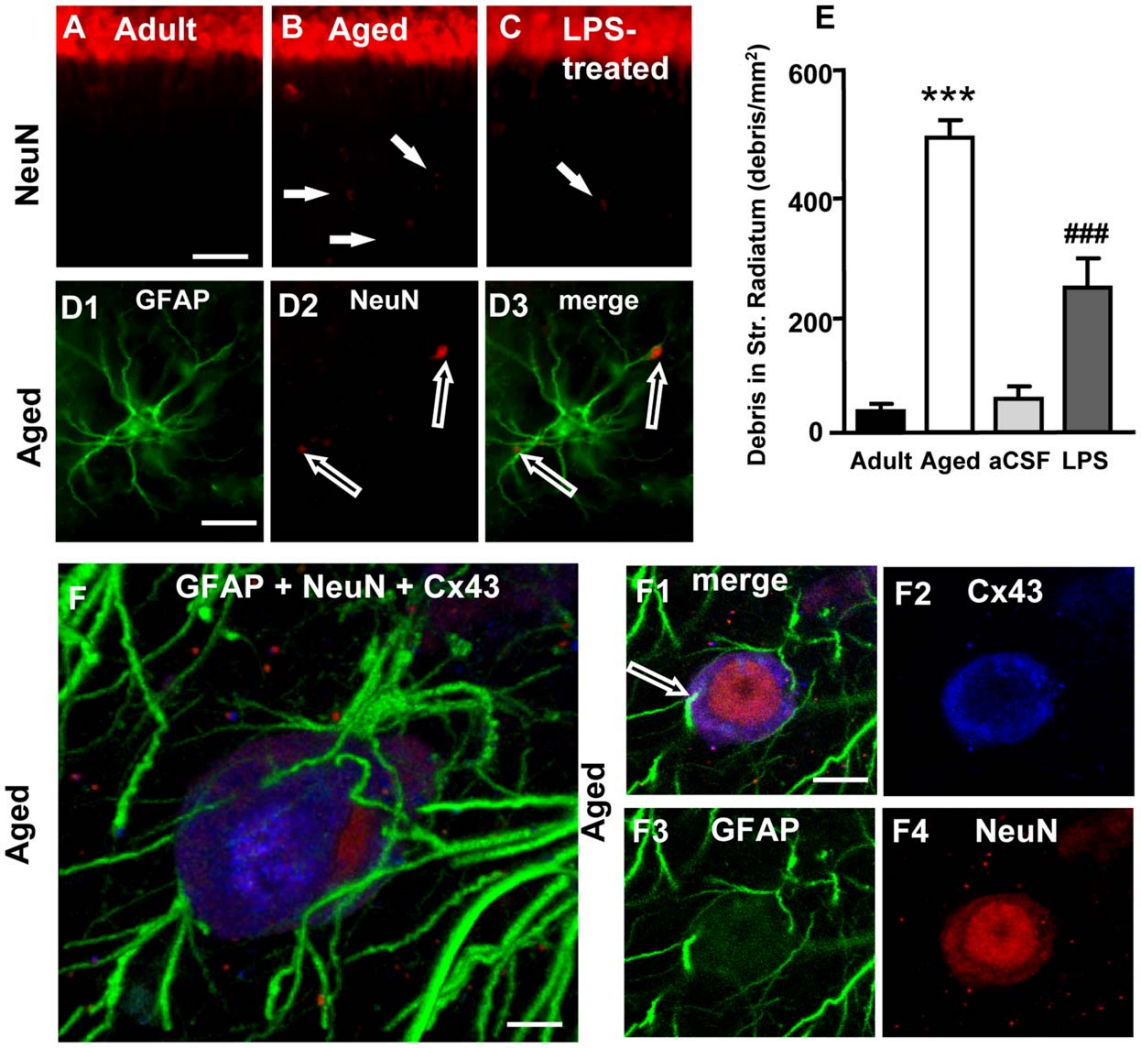
Quantitative analysis of neuronal debris in Str. Radiatum, involvement of Cx43 in astrocytes-neuron interplay. Images from CA1 Str. Pyramidalis and CA1 Str. Radiatum of an adult (**A**), aged (**B**) and LPS-treated rat (**C**) showing the presence of neuronal debris (arrows, **B** and **C**). Scale bar: 70 µm. **D1–D3:** higher magnification images of GFAP (green, **D1**) and NeuN (red, **D2**) staining and the merge of the two previous images (**D3**). Empty arrows show neuronal debris closely apposed to astrocyte branches. Scale bar: 15 µm**. E:** quantitative analysis of neuronal debris in CA1 Str. Radiatum of adult (n = 12), aged (n = 10), aCSF- (n = 5) and LPS-treated (n = 6) rats (mean±SEM; *** and ^###^P<0.001 vs all other groups). **F–F4**: Representative images of triple immunostaining of GFAP (green), NeuN (red) and Cx43 (blue) in the Str. Radiatum of an aged rat. **F**: 3D stack of 39 confocal scans (total 14.39 µm); **F1**: a “sub-slice” of the previous neuron (obtained stacking 6 consecutive scans, total 1.843 µm, starting at a depth of 5.899 µm into the cell) and separate staining of Cx43 (**F2**), GFAP (**F3**) and NeuN (**F4**). Scale bar: 5 µm (**F)**; 10 µm (**F1–F4).**

Colocalization of different antigens was performed using combinations of different primary and secondary antibodies, as reported in Results section, followed by double or triple labeling confocal microscopy.

Nuclei were stained using DAPI (Vectashield hard set with DAPI, Vector Laboratories).

#### Light microscopy immunohistochemistry Day 1

The sections were stained using a conventional free-floating method as described [13].

#### Day 1

Coronal brain slices were placed in wells of 24-well plates and were rinsed for 10 min in phosphate buffered saline (PBS) 0.3% Triton X-100 (PBS-TX), incubated for 15 min in PBS-TX containing 0.75% H_2_O_2_ and blocked with blocking buffer (BB) containing 1.5% normal goat serum and 0.05% NaN_3_ in PBS-TX for 1 h. Slices were then incubated overnight at 4°C with the primary antibody anti-GFAP.

**Figure 7:**
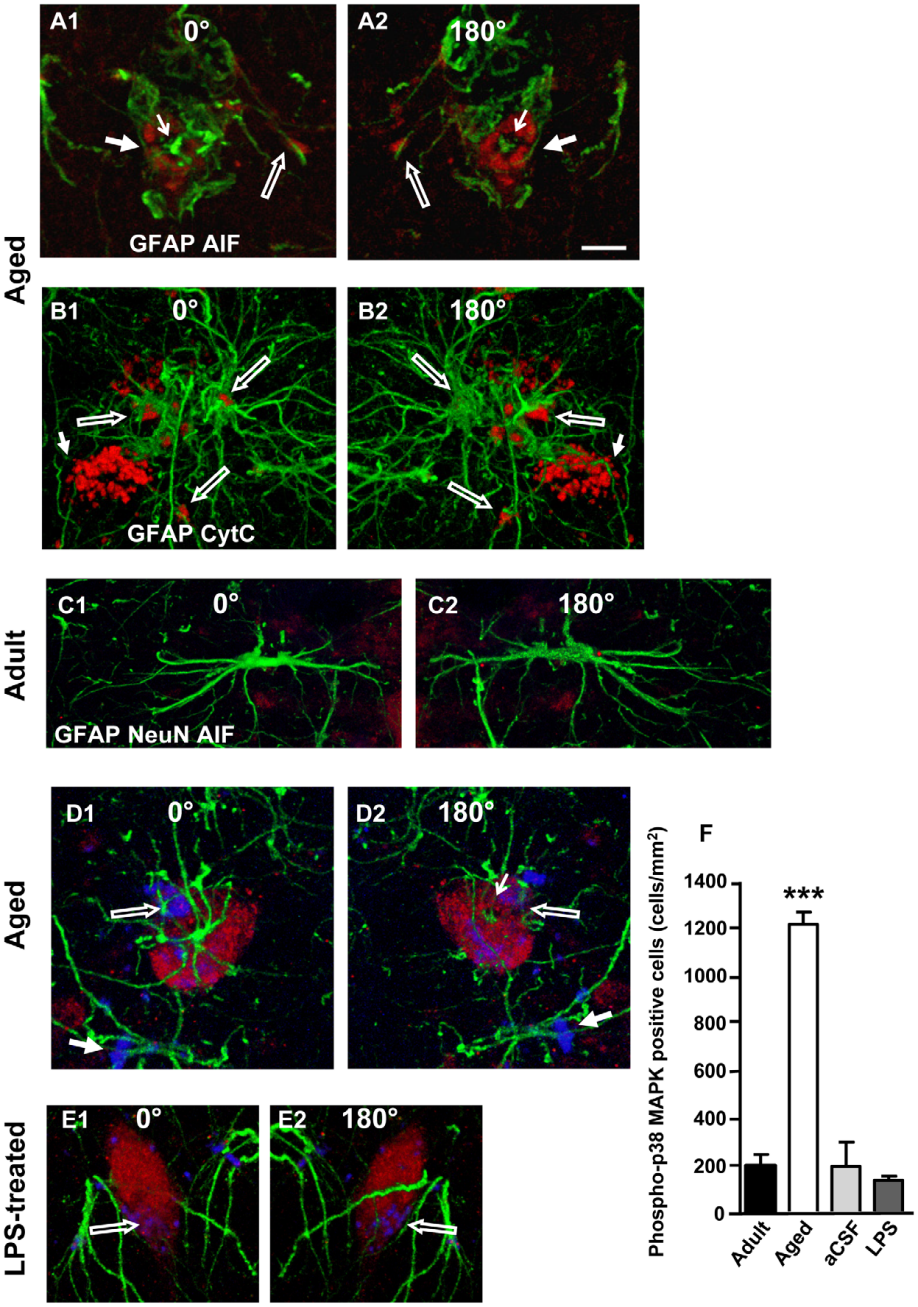
Immunostaining of markers of apoptosis in cells surrounded by astrocyte branches in CA1 Str. Radiatum. **A1,A2:** immunostaining for CytC (red) and GFAP (green). **B1,B2:** immunostaining for AIF (red) and GFAP (green). µm. Representative images of immunostaining for NeuN (red), AIF (blue) and GFAP (green) taken from the CA1 region of an adult (**C1–C2**), an aged (**D1–D2**) and an LPS-treated (**E1–E2**) rat. Note the presence of AIF staining within neurons of aged and LPS treated rats only (open arrows in **D1,D2** and **E1,E2**). This effect was observed in all slices from aged and LPS-treated rats. Scale bar: 10. **F:** Quantification of phospho-p38MAPK positive cells in CA1 Str. Pyramidalis of adult (n = 11), aged (n = 16), aCSF- (n = 5) and LPS-treated (n = 5) rats (mean±SEM; ***P<0.001, vs all other groups).

#### Day 2

Slices were incubated for 2 h at RT in secondary antibody diluted in BB, then for 1 h 30 min in AB solution (Vectastain ABC kit, Vector Laboratories); staining was developed for 2–3 min using 3,3′-diaminobenzidine (DAB) staining kit (Vectastain, Vector Laboratories) with NiCl_2_ as an enhancer. DAB-stained slices were examined using an Olympus BX40 microscope equipped with an Olympus DP 50 (Olympus, Milan, Italy) digital camera.

**Figure 8:**
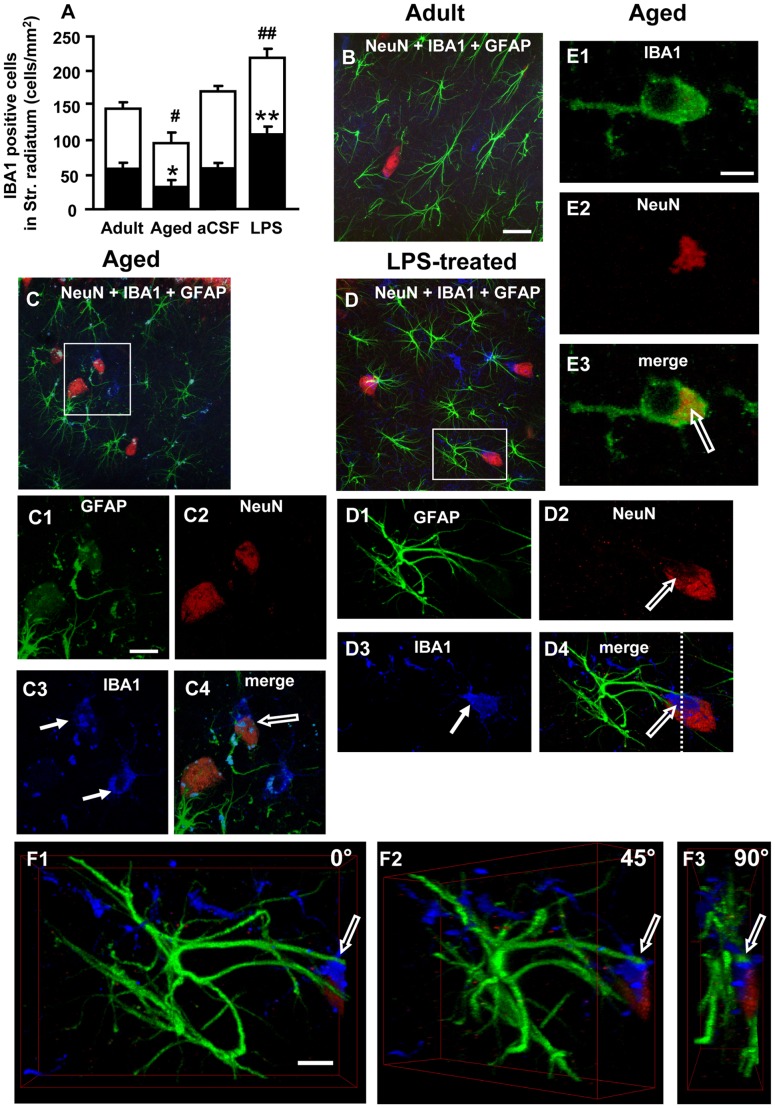
Quantitative analysis of resting-reactive microglia cells, 3D-analysis of neuron-astrocyte-microglia interplay. A: quantitative analysis of microglia cells (mean±SEM) in CA1 Str. Radiatum of adult (n = 12), aged (n = 12), aCSF- (n = 5) and LPS-treated (n = 6) rats. Black portion of columns represent reactive microglia cells, white portion of columns represent resting microglia cells, the entire columns represent total microglia cells; *at least P<0.05 vs all other groups; **P<0.01 vs all other groups; #at least P<0.05 vs all other groups; ##P<0.01 vs all other groups. **B,C,D**: Merged confocal images of triple immunostaining of neurons (NeuN, red), astrocytes (GFAP, green) and microglia (IBA1, blue) in the CA1 Str. Radiatum of adult (**B**), aged (**C**), and LPS-treated rats (**D**). Scale bar: 20 µm. **C1–C4** and **D1–D4**: higher magnification confocal images of framed areas in **C** and **D**, respectively. Arrows in **C3** and **D3** indicate IBA1-positive reactive microglia cells. Open arrows in **C4** and **D4** show microglia cells phagocytising neurons. Scale bar: 7 µm. **E1–E3:** a confocal “sub-slice” (thickness 0.7 µm) acquired at depth 2.8 µm into a microglia cell (IBA1-positive, green) from the Str. Radiatum of an aged rat showing complete co-localization of a neuronal debris (**E2**, red, immunostained for NeuN) in the microglia cytoplasm (**E3**, yellow-orange, open arrow). Scale bar: 7 µm. **F1–F3∶**3D stacks of the neuron shown in **D4**, digitally cut along the white dotted line and rotated by 0, 45 and 90 degrees along the vertical axis. Scale bar: 10 µm.

#### Fluorescent immunohistochemistry

Immunostaining was performed on coronal slices with the free-floating method [14]. For further details see also *Antibodies and Methodological considerations*.

#### Day 1

For double immunostaining, free-floating slices (40 µm thick) were placed in wells of 24-well plates and were rinsed for 10 min in PBS-TX and blocked for 60 min with BB containing 10% Normal Goat Serum - 10% Normal Horse Serum in PBS-TX and 0.05% NaN_3_. Slices were then incubated overnight at 4°C under slight agitation with a combination of two primary antibodies, both dissolved in BB. The following primary antibodies were used: a mouse monoclonal anti-neuronal nuclei (NeuN, 1∶200; Chemicon, Temecula, CA, USA) for neurons; a rabbit polyclonal anti-glial fibrillary acidic protein (GFAP, 1∶1000; DakoCytomation, Glostrup, Denmark) for astrocytes and a rabbit polyclonal IBA1 (1∶300, WAKO Pure Chem. Ind, Osaka, Japan) for microglia; a mouse monoclonal against Cytochrome C (1∶200, Becton and Dickinson, Franklin Lakes, NJ, USA); a goat polyclonal against the Apoptosis Inducing Factor (AIF, 1∶100, Santa Cruz Biotechnology, Santa Cruz, CA, USA); a rabbit polyclonal against CX3CL1 (1∶400, Abcam, Cambridge, UK).

**Figure 9:**
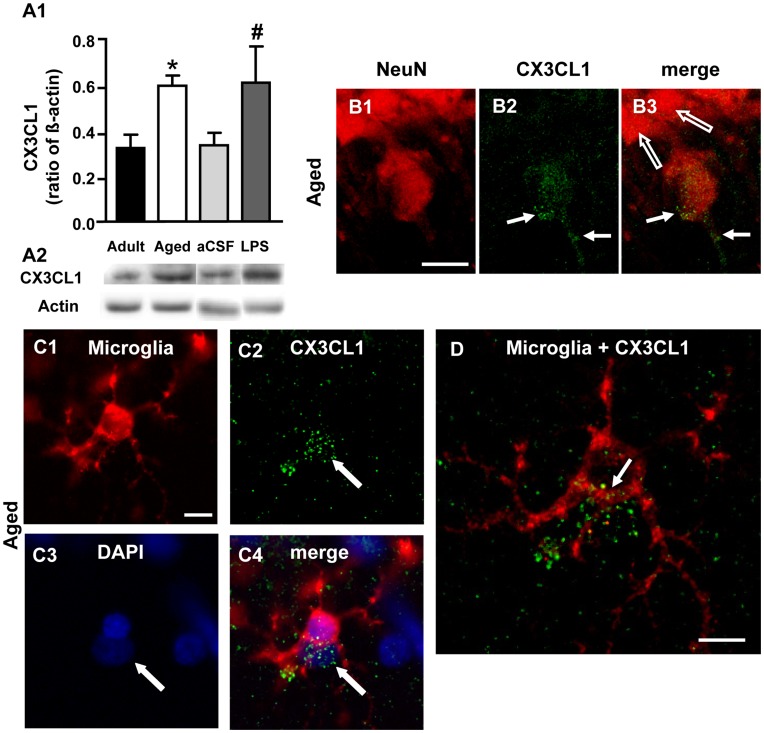
Analysis of CX3CL1 expression in the hippocampus of adult, aged, aCSF- and LPS-treated rats. **A1**: Western Blot analysis of CX3CL1 in whole hippocampus homogenates of adult (n = 7), aged (n = 4), aCSF- (n = 4) and LPS-treated (n = 4) rats. Each column in the graph represents the level of CX3CL1 expressed as mean±SEM, normalized to β-actin run in the same gel. *P<0.05 vs adult; #P<0.05 vs aCSF-tretated. **A2:** representative Western Blot runs of CX3CL1 and of β-actin. **B1–B3:** laser confocal microscopy immunohistochemistry of neurons (**B1,** NeuN, red), CX3CL1 (**B2,** green) and the merge of the two previous images (**B3**) from the CA1 region of an aged rat. Scale bar: 14 µm. **C1–C4:** epifluorescent microscopy images of a microglia cell (**C1**, red), CX3CL1 immunostaining (**C2,** green), DAPI staining of nuclei (**C3,** blue) and the merge of the three previous images (**C4**), indicating that CX3CL1 staining is localized on the surface of a cell, possibly a neuron (arrows). **D:** the image represents a confocal “sub-slice” (total thickness 2.233 µm) of the same microglia cell shown in C1–C4, acquired starting at a depth of 8.932 µm into the cell. The CX3CL1 positive cell (green) is partially colocalized with the microglia cell. Scale bar: 14 µm.

**Figure 10:**
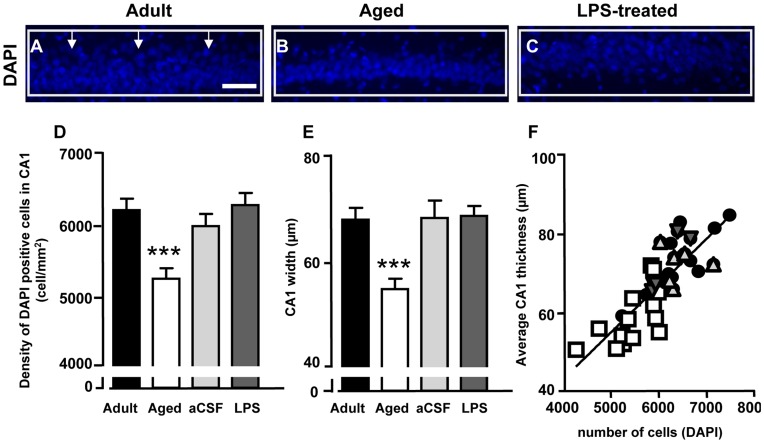
Quantitative analysis of CA1 pyramidal nuclei labelled with DAPI. **A–C:** representative photomicrographs of DAPI staining in CA1 Str. Pyramidalis of adult (**A**), aged (**B**) and LPS-treated (**C**) rats. DAPI positive nuclei were counted within the framed areas, and thickness of CA1 Str. Pyramidalis was measured in correspondence to the vertical arrows. Scale bar: 60 µm. **D:** quantitative analysis of DAPI positive nuclei in CA1 Str. Pyramidalis of adult (n = 14), aged (n = 14), aCSF- (n = 5) and LPS-treated (n = 6) rats (cells/mm^2^, mean±SEM; ***P<0.01 vs all other groups). **E:** quantitative analysis of CA1 Str. Pyramidalis thickness of adult (n = 14), aged (n = 14), aCSF- (n = 5) and LPS-treated (n = 6) rats (mean±SEM). ***P<0.001 vs all other groups. **F**: linear regression analysis of CA1 pyramidal cells vs CA1 thickness; black circles: adult rats; light grey triangles: aCSF-treated; dark grey triangles: LPS-treated rats; white squares: aged rats.

For triple immunostaining, free-floating slices (40 µm thick) were placed in wells of 24-well plates and were rinsed for 10 min in PBS-TX and blocked for 60 min with BB containing 10% Normal Goat Serum - 10% Normal Horse Serum in PBS-TX and 0.05%.

NaN_3_. Slices were then incubated overnight at 4°C under slight agitation with a combination of two primary antibodies: a mouse monoclonal anti-neuronal nuclei (NeuN, 1∶200; Chemicon, Temecula, CA, USA) for neurons and one of the following primary antibodies: a rabbit polyclonal antibody against Cx43 (1∶50, Santa Cruz Biotechnology), or a goat polyclonal against AIF (1∶100, Santa Cruz Biotechnology, Santa Cruz, CA, USA), or a rabbit polyclonal against IBA1 (1∶300, WAKO Pure Chem. Ind, Osaka, Japan) for microglia.

#### Day 2

For double immunostaining, slices were incubated for 2 h at room temperature in the dark with AlexaFluor 488 conjugated donkey anti-rabbit IgG (1∶400, Invitrogen Life Sciences, Carlsbad, CA, USA) secondary antibody diluted in BB and then for 2 h at room temperature in the dark with AlexaFluor 488 conjugated donkey anti-rabbit IgG secondary antibody plus AlexaFluor 594 goat anti mouse both diluted 1∶400 in BB.

**Table 1:** Summary of results.

	Adult	Aged% vs adult	aCSF% vs adult	LPS% vs adult
**GFAP levels**	0.65±0.17	**+108%**	+11%	**+152%**
**GFAP positive cells**	522.0±8.2	**−20%**	+0.1%	+5%
**Astrocyte branches**	33.3±1.1	**−15%**	−4%	−2%
**Debris**	35.61±12.6	**+1294%**	+58%	**+585%**
**Ph-p38MAPK positive cells**	203.2±40.7	**+500%**	−4%	−33%
**Total microglia**	127.3±12.2	−**26%**	+12%	**+70%**
**Reactive microglia**	53.78±10.3	−**42%**	+7%	**+98%**
**Resting microglia**	73.47±8.4	−**15%**	+49%	**+49%**
**CX3CL1 levels**	0.32±0.06	**+84%**	+3%	**+91%**
**DAPI in CA1**	6272±148.2	−**14%**	−3%	+1%
**CA1 thickness**	71.3±2.2	−**18%**	+1%	+1%

The first data column reports the data obtained from the quantitative analyses performed in adult rat hippocampus, taken as controls: GFAP levels in hippocampal homogenates (ratio of ß-actin); GFAP positive cells in CA1 Str. Radiatum (number); Astrocyte branches in CA1 Str. Radiatum (µm); Debris in CA1 Str. Radiatum (number); Phospho-p38MAPK positive cells in CA1 Str. Pyramidalis (number); Total microglia in CA1 Str. Radiatum (number); Reactive microglia in CA1 Str. Radiatum (number); Resting microglia in CA1 Str. Radiatum (number); CX3CL1 levels in hippocampal homogenates (ratio of ß-actin); DAPI in CA1 Str. Pyramidalis (number); CA1 Str. Pyramidalis thickness (µm). All other data are reported as percent variations of those found in adult rats.

For triple immunostaining, slices were incubated for 2 h at room temperature in the dark with AlexaFluor 555 donkey anti mouse IgG (1∶400) secondary antibody diluted in BB and then for 2 h at room temperature in the dark with AlexaFluor 555 donkey anti mouse IgG (1∶400) plus either one of the following secondary antibodies: AlexaFluor 633 donkey anti-goat IgG (1∶400) or Alexa Fluor 635 goat anti-rabbit (1∶400) as necessary, depending upon the primary antibodies used. After washings, GFAP was immunostained using the fluorescent primary antibody AlexaFluor 488.

For both double and triple immunostaining procedures, after extensive washings slices were mounted onto gelatin-coated slides using Vectashield hard set with DAPI (Vector Laboratories) as a mounting medium.

Slices were observed under an epifluorescent microscope Olympus BX40 equipped with an Olympus DP 50 digital camera (Olympus, Milan, Italy) or under a LEICA TCS SP5 confocal laser scanning microscope (Leica Microsystems CMS GmbH, Mannheim, Germany). Confocal scans were taken at 0.349 µm z-steps keeping all parameters (pinhole, contrast and brightness) constant. Image analyses were conducted on image z-stacks which contained the field of interest. Images were digitally converted to green, to red or blue using Image J (National Institute of Health, http://rsb.info.nih.gov/ij) and digitally combined to obtain single, double or triple labelled images and then assembled into montages using Adobe Photoshop (Adobe Systems, Mountain View, CA, USA). High power 3D z-stacks of the images were obtained using Image J 3D viewer. Movies were obtained by recording the 360° rotations of the 3D stacks obtained using Image J 3D viewer.

#### Methodological considerations

All quantification analyses were performed blind by two different experimenters and results were averaged. Three coronal slices (spaced by 150 µm, starting at about −2.8 mm from bregma) containing the dorsal hippocampus were immunostained. The region of interest (ROI) in CA1 containing Str. Pyramidalis and Str. Radiatum was consistently captured at 20x magnification using an Olympus digital camera (framed area in Figure 1). Areas of ROI in CA1 were calculated in mm^2^ and the counts of immunopositive cells expressed as number of cells/mm^2^. Astrocytes and cell debris in CA1 Str. Radiatum as well as neurons of pyramidal CA1 were counted choosing the same area in all slices used (see framed areas in Figure 1) and counts were expressed as number of cells/mm^2^. The length of principal astrocyte branches (chosen randomly) was measured using Image J. Two independent experimenters measured 4 principal branches of three astrocytes per ROI and results were averaged. Cell debris was defined as NeuN-positive fragments with dimensions ranging between 2.5 and 6.5 µm. The criteria to characterize a microglia cell as “resting” or “reactive” were defined before evaluation as follows. In accordance to the literature [15]–[19] resting microglia were defined as cells with small,round cell bodies with thin and highly ramified branches equally distributed around the cell body (Figure 1C). Reactive microglia were defined as cells with a pleomorphic bi-or tri-polar cell body, (D) or as spindle or rod-shaped cells (E,F) both with modification in cellular structure that included de-ramification as well as shortening and twisting of cellular processes (Figure 1 D,E,F). All reactive microglia cells had bigger cell bodies than resting microglia. In order to evaluate the thickness of the CA1 pyramidal cell layer, three measurements were taken at three locations evenly distributed throughout the chosen ROI (arrows in Figures 1A and 10A) and then averaged.

Triple immunostaining for different cell markers was restricted by the commercial availability of primary antibodies raised in different species and the proper secondary antibodies. In some instances GFAP was immunostained using a fluorescent (AlexaFluor 488) primary antibody. AlexaFluor 633 secondary antibodies were used in confocal triple immunostaining and converted to a false blue colour (Figure 8). Images with DAPI were taken under an epifluorescent microscope. In order to unambiguously define whether astrocyte branches were infiltrating neurons we “sub-sliced” the neuron by stacking few consecutive confocal z-scans acquired at different depths throughout the cell body or we rotated the 3-d stack around the cell vertical axis.

Immunohistochemical staining controls were performed by omitting the primary or secondary antibodies to confirm the specificity of the staining.

### Western Blot

The following antibodies were used: anti-CX3CL1 (rabbit polyclonal, 1∶300; Abcam), anti-actin (rabbit polyclonal, 1∶10,000; Sigma Chem Co., St. Louis, MO, USA), anti-GFAP (rabbit polyclonal, 1∶30,000, BD Biosciences, Franklin Lakes, NJ, USA). WB was carried out as previously described [13].

### Statistical Analyses

All quantitative analyses were performed blind to the experimental conditions by two different experimenters and their data averaged. Statistical comparisons were performed using Student’s t test, one way ANOVA followed by Newman-Keuls multiple comparison test (if more than two groups were compared), or two-way ANOVA followed by Bonferroni post-hoc test (if two variables were compared), as appropriate. Correlation analysis was performed using the linear regression analysis and r^2^ was generated. All statistical analyses were performed using Graph Pad Prism (Graph Pad Software Inc., La Jolla, CA, USA). Significance was set at P<0.05.

## Results

### Astrocytes in the CA1, CA3 and Dentate Gyrus of Adult, Aged, aCSF- and LPS-treated Rats

Western blot analysis of GFAP, a prototypical marker for astrocytes, in whole hippocampus homogenates of adult, aged, aCSF- and LPS-treated rats showed that GFAP protein expression increased significantly by about 108% in aged rat hippocampus as compared to the adult rats and by about 129% in LPS-treated rat hippocampus in comparison to aCSF-treated rat (one way ANOVA: F_(3,17)_ = 4.565; P = 0.0160; *P<0.05 vs adult and # P<0.05 vs aCSF-treated rats, Newman-Keuls multiple comparison test, Figure 2A1).

Quite unexpectedly, in the first series of experiments we found that astrocytes immunostained for GFAP with DAB were significantly less numerous in CA1, CA3 and DG of aged rats in comparison to adult rats (Figure 2B–C3). Indeed, in the adult CA1 Str. Radiatum we found that the number of GFAP positive cells/mm^2^ was 594.8±19.8 as compared to 407.8±25.7 in the aged CA1 Str. Radiatum (−22%, P<0.0001, Student’s t test). Similarly, in the adult CA3 Str. Radiatum the number of GFAP positive cells/mm^2^ was 517.7±22.3, as compared to 325.3±15.2 in the aged CA3 Str. Radiatum (−27%, P<0.0001, Student’s t test). Finally, in the adult DG we found 533.8±17.8 GFAP positive cells/mm^2^ as compared to 427.8±29.4 GFAP positive cells/mm^2^ in the aged DG (−20%, P<0.01, Student’s t test). For comparison, we also measured GFAP positive cells/mm^2^ in the pyriform cortex, a brain area involved in olfactory memory. No significant difference was found among the three experimental groups. The number of GFAP positive cells/mm^2^ in the pyriform cortex of adult rats was 428.4±28.5 (n = 5), in aged rats was 436.5±9.7 (n = 5) and in LPS-treated rats was 435.1±3.4 (n = 5).

### Quantification of Astrocytes in the Hippocampus of Adult, Aged, aCSF- and LPS-treated Rats with Fluorescent Immunohistochemistry

On the basis of these unexpected findings between aged and adult rats we then further characterized astrocytes in the pyramidal cell layer and Str. Radiatum of CA1 (ROI, Figure 1A) of normal aged and LPS-treated rats in comparison to normal adult rats and aCSF-treated rats, respectively, and studied the mutual interactions of astrocytes with neurons and microglia. Astrocytes were immunostained for GFAP using a fluorescent secondary antibody and counterstained with DAPI. Figure 3 shows representative images of GFAP (green) and DAPI (blue) staining in CA1 Str. Pyramidalis and CA1 Str. Radiatum of adult (A1,A2,A3) and aged (B1,B2,B3) rats. Quantitative analysis performed with this different technique on adult, aged, aCSF- and LPS-treated rat slices, again demonstrated that in CA1 Str. Radiatum of aged rats the number of astrocytes/mm^2^ was significantly lower (by 20%) as compared to adult rats (Figure 3C, one way ANOVA: F_(3,35)_ = 11.12; P<0.0001; **P<0.01 vs all other groups, Newman-Keuls multiple comparison test), while no significant differences were observed in LPS-treated rats.

Quantitative analysis of astrocytes branch length was performed (see methods, Figure 1B); the results are shown in Figure 3D. The length of principal branches was significantly shorter in aged rats as compared to adult, aCSF- and LPS-treated rats (−15% vs adult rats; one way ANOVA: F_(3,35)_ = 6.055; P = 0.002; **P<0.01 vs all other groups, Newman-Keuls multiple comparison test). Images taken at higher magnification (Figure 4) demonstrate that in the hippocampus of aged rats astrocytes appear smaller than adult, aCSF- (not shown) and LPS-treated rats. Panels A1–A4, B1–B4 and C1–C4 of Figure 4 show 3D reconstructions of single astrocytes from adult, aged and LPS-treated rats, respectively, acquired and z-stacked using the confocal software and rotated by 60, 120 and 180 degrees around its vertical axis. These images demonstrate the altered morphology of aged rat astrocytes (B1–B4) which have shorter principal branches that appear twisted and bent compared to adult (A1–A4) and LPS-treated rats (C1–C4). At closer examination, aged astrocytes appeared to have lost their most distal principal processes which have a highly fragmented aspect, a modification termed clasmatodendrosis by Cajal [20].

### Characterization of Astrocyte-neuron Interactions in the Hippocampus of Adult, Aged and LPS-treated Rats with Fluorescent Immunohistochemistry

Images of double immunostaining of astrocytes with GFAP antibody (green) and neurons with NeuN antibody (red) in the CA1 Str. Pyramidalis and CA1 Str. Radiatum of adult (A), aged (B) and LPS-treated rat (C) are shown in Figure 5. In the CA1 Str. Radiatum of aged and LPS-treated rats several neurons can be seen to be surrounded by astrocyte branches which were in close proximity to the cell (D), embracing and apparently wedging them to form smaller cellular fragments called “debris”. A higher magnification image showing this intimate interrelationship between astrocyte branches and neuronal cell bodies is shown in D. Astrocyte branches are intermingled within the cell body of the neuron. In order to further characterize this phenomenon, we “sub-sliced” the neuron shown in Figure 5D into three serial confocal images acquired throughout the cell body. We obtained three “sub slices” of the cell by stacking two consecutive confocal scans (total 0.738 µm) spaced 1.845 µm from one another (E1 was acquired at 2.209 µm, E2 at 4.792 µm, and E3 at 7.375 µm depth into the cell). The digitally combined images of GFAP and NeuN staining show that astrocyte branches are present not only on the neuron surface, but also inside the neuron body; for example, they are visible in cell “sub-slices” obtained from the inside of the cell (empty arrows in E2 and E3). The cell confocal 3D stack of the neuron shown in D was digitally cut along the dotted white line (D), as shown in F1, and the image was digitally rotated by 45 (F2) and 90 (F3) degrees around its vertical axis to further visualize the inside of the neuron. It is clearly visible from panels E1–E3 and F2–F3 that a portion of an astrocyte branch resides inside the neuronal cell body (open arrows). A further demonstration of the astrocyte-neuron interaction can be appreciated in Movie S1.

Neurons associated with astrocytes in this intimate manner show signs of degeneration, such as lack of the nucleus, which is a typical characteristic of cells undergoing apoptosis. These morphological modifications are consistent with the hypothesis that astrocytes are bisecting a dying (apoptotic) neuron into neuronal debris (further proof is provided below in the next paragraph). In LPS-treated rats, this phenomenon was less frequent; in CA1 Str. Radiatum of adult rats it was never observed.

NeuN staining revealed the presence of neuronal debris scattered throughout the CA1 Str. Radiatum of aged and LPS-treated rats (arrows in Figure 6B and C). In the GFAP and NeuN double stained slices neuronal debris always appeared closely apposed to astrocyte cell bodies or branches (empty arrows in Figure 6D2 and D3). A quantitative analysis of neuronal debris (Figure 6E) showed that in the CA1 Str. Radiatum of aged and LPS-treated rats neuronal debris was significantly more numerous than in adult and aCSF-treated rats (one way ANOVA F_(3,29)_ = 108.1; P<0.0001; ***P<0.001 and^ ###^P<0.001 vs all other groups, Newman-Keuls multiple comparison test).

Some slices (n = 3 per group) were triple immunostained for GFAP (green), NeuN (red) and Cx43 (blue) (Figure 6F–F4) to perform a qualitative analysis of an intercellular protein (Cx43) that might be responsible for the interconnection between astrocytes and neurons. Strikingly, we found that neurons that were in intimate contact with astrocytes in the hippocampus of aged rats expressed high levels of Cx43. Panel F shows a 3D stack of a neuron localized in the CA1 Str. Radiatum of an aged rat; panel F1 represents a “sub-slice” obtained from the same neuron stacking 6 consecutive scans (total thickness 1.843 µm) starting at a depth of 5.899 µm into the cell. This image clearly shows that astrocytes branches intermingle inside the cell (empty arrow) and that Cx43 is highly expressed in the cytoplasm in proximity to the plasma membrane (F2) and close to the astrocyte branches. This effect was observed in all slices analyzed from aged and LPS-treated rats.

### Apoptosis Increases in the Hippocampus of Aged and LPS-treated Rats

Hippocampal slices from adult, aged, aCSF- and and LPS-treated rats (n = 3 per group) were double immunostained with anti-GFAP for astrocytes (green) and, separately, with one of the following markers for apoptosis, namely CytC (Figure 7A1,A2) or AIF (Figure 7B1,B2), in order to verify whether neurons closely apposed to astrocytes were undergoing apoptosis and whether cellular debris “trapped” by astrocytes branches were apoptotic fragments. Panels A1 and A2 represent 3D stacks of 40 confocal scans (total thickness 14.76 µm) while panels B1 and B2 represent 3D stacks of 59 confocal scans (total thickness 21.77 µm) taken in the CA1 Str. Radiatum of aged rats. To better demonstrate the mutual interactions between astrocyte branches, apoptotic neurons and cell debris, images in A1–A2 and B1–B2 are views of the 3D stacks rotated by 180 degrees (A1 and B1 show a “top-down view” while A2 and B2 show a “bottom-up view” of the 3D stacks). In aged rats as well as LPS-treated rats (not shown) cells, identified as neurons by their shape and location, were positive for either CytC (red, Figure 7A1 and A2) or AIF (red, Figure 7B1 and B2) and were seen flanking astrocyte branches; these branches were in intimate contact with the neuronal membrane (thick arrows). In A1 and A2 it is evident that the cell nucleus is missing and the astrocyte branches are bisecting the neuron (thin arrows). Cellular debris positive for the apoptotic markers were also in close contact with astrocyte branches (open arrows). From the 3D stack of the cells shown in B1,B2 we obtained Movie S2 which further shows the interaction between astrocyte branches and Cyt C-positive cells and debris.

We further characterized the apoptotic staining and demonstrated that AIF positive cells were neurons via triple immunostaining for neurons (NeuN, red), astrocytes (GFAP, green) and AIF (AIF, blue) (Figure 7 C1–C2,D1–D2,E1–E2). No evidence of apoptotic neurons was found in CA1 pyramidal cell layer and CA1 Str. Radiatum of adult rats and aCSF-treated rats (not shown). Panels C1 and C2 represent 3D stacks of 38 confocal scans (total thickness 13.77 µm) taken in the CA1 Str. Radiatum of adult rats, panels D1 and D2 represent 3D stacks of 59 confocal scans (total thickness 21.38 µm) taken in the CA1 Str. Radiatum of aged rats, while panels E1 and E2 represent 3D stacks of 33 confocal scans (total thickness 11.98 µm) taken in the CA1 Str. Radiatum of LPS-treated rats (as above, C1, D1 and E1 show a “top-down view” while C2, D2 and E2 show a “bottom-up view” of the 3D stacks). Neurons positive for AIF (open arrows) were closely surrounded by astrocyte branches (E4 and F4). Complete co-localization of NeuN with anti-AIF immunostaining was consistently present in cellular debris attached to astrocyte branches (D1 and D2, arrows). Immunostaining for NeuN, GFAP and CytC showed similar results as above in all experimental groups (data not shown). This effect was observed in all slices analyzed from aged and LPS-treated rats.

We also performed immunostaining of phospho-p38MAPK, a transduction pathway with many different roles related to cell stress and apoptosis. Quantitative analysis (Figure 7F) found a statistically significant increase in the number of phospho-p38MAPK positive neurons in the CA1 Str. Pyramidalis of aged rats, as compared to all other groups (one way ANOVA F_(3,33)_ = 103.1; P<0.0001; ***P<0.001 vs all other groups, Newman-Keuls multiple comparison test). Such an increase was not found in acsf- or LPS-treated rats. Neither ageing nor LPS treatment activated JNK (not shown).

### Reactive Microglia in the Hippocampus of Adult, Aged, aCSF- and LPS-treated Rats

Immunostaining and quantification of both resting and reactive microglia cells in the CA1 Str. Radiatum of the four experimental groups was performed using the selective IBA1 antibody. Immunostaining for neurons (NeuN, red), astrocytes (GFAP, green) and microglia (IBA1, blue) in the CA1 Str. Radiatum of adult (B), aged (C, C1–C4), and LPS-treated rats (D,D1–D4) is shown in Figure 8. Each column in Figure 8A, subdivided into reactive microglia (black) and resting microglia (white), represents the total microglia (cells/mm^2^) in the CA1 Str. Radiatum of the four experimental groups. A significant difference was found among the four experimental groups (Two-way ANOVA: Age/Treatment: F_(3,126)_ = 17.88; P<0.0001; microglia state: F_(2,126)_ = 38.36; P<0.0001; Interaction: F_(6,126)_ = 3.65_,_ n.s.). The total number of microglia significantly decreased (^#^P<0.05 vs all other groups, Bonferroni post-hoc test) in CA1 Str. Radiatum of aged rats while it was increased in CA1 Str. Radiatum of LPS-treated rats (^##^P<0.01 vs all other groups; Bonferroni post-hoc test). Most interestingly, significant differences among the four experimental groups were mainly found in the fraction of reactive microglia, which significantly decreased in aged rats (*P<0.05 vs all other groups, Bonferroni post hoc test) and significantly increased in LPS-treated rats (**P<0.01 vs all other groups, Bonferroni post-hoc test). In contrast, the total number of resting microglia was not significantly different among the four experimental groups. Although the quantity of microglia cells in the CA1 Str. Radiatum of aged rats was decreased, the microglia were still actively involved in phagocytosis. Indeed, reactive microglia, phenotipically carachterized according to the literature (discussed in *Methodological considerations*) were present in the CA1 Str. Radiatum of aged and LPS-treated rats (arrows in C3 and D3). For example, high magnification confocal images in C1–C4 and in D1–D4 for aged and LPS-treated rats, respectively, show (open arrows) that reactive microglia (blue) are phagocytosing neurons (red).In Figure 8E1–E3 a microglia cell “sub-slice,” acquired at 2.8 µm depth into the cell (total thickness 0.7 µm) shows NeuN-positive neuronal debris (red, E2) completely internalized within the microglia cytoplasm (orange-yellow, E3, open arrow).

It also appears that an intercellular communication among astrocytes, microglia and neurons is taking place by recruitment and activation of different glial cells in a well-organized reciprocal interaction. The cell confocal 3D stack of the neuron-microglia-astrocyte “triad” shown in D4 was digitally cut along the dotted white line, as shown in F1, and the image was digitally rotated by 45 (F2) and 90 (F3) degrees around its vertical axis to better visualize the astrocyte- microglia-neuron interaction. It appears clearly visible from panels F2 and F3 and from Movie S3 that the microglia cell (blue) is in close contact and possibly starts to phagocytose the neuronal cell body (red), as also demonstrated in panel D2 where part of the cytoplasm underneath the microglia cell is clearly missing (open arrow in D2). An astrocyte branch resides on top of the microglia and fully in contact with it (open arrows). Movies S4 and S5 show two other astrocytes-microglia-neuron “triads” that further demonstrate this point.

### Increased CX3CL1 Expression in CA1 of Aged and LPS-treated Rats

WB analysis of the protein CX3CL1 in whole hippocampus homogenates of adult, aged, aCSF- and LPS-treated rats showed that CX3CL1 expression increased significantly by about 84% in aged rat hippocampus in comparison to adult rats and by about 85% in LPS-treated rat hippocampus in comparison to aCSF-treated rats (one way ANOVA: F_(3,15)_ = 3.478; P = 0.0427; *P<0.05 vs adult; # P<0.05 vs aCSF-treated Newman-Keuls multiple comparison test, Figure 9A1). Laser confocal microscopy of NeuN (B1, red) and CX3CL1 (B2, green) obtained from a “sub-slice” acquired at 7.329 µm depth into the neuron stacking ten confocal scans (total 3.49 µm) shows that CX3CL1 immunostaining increased in the cell body and apical dendrite (B2,B3, arrows) of some but not all neurons (B3, open arrows) of the CA1 area of aged rats. Immunostaining of microglia (C1, red), CX3CL1 (C2, green) and nuclei with DAPI (C3, blue) showed that CX3CL1 was highly expressed in a cell (demonstrated by co-localization with DAPI, arrows, C2,C3,C4) which was localized in close contact with a microglia cell, as also demonstrated by DAPI staining in C3 (arrow). Indeed, the confocal image in Figure 9D obtained from a “sub-slice” of the same microglia cell shown in C1–C4, acquired at 8.932 µm depth into the cell, stacking eight confocal scans (total 2.233 µm), clearly shows that the green immunostaining of CX3CL1 is partially superimposed with the microglia cell, thus indicating that phagocytosis of the CX3CL1-positive cell is taking place (arrow). This image, taken together with those presented in B1–B3 and C1–C4, and from data of the literature, indicates that CX3CL1 immunostaining is localized on a neuron which is being phagocytised by the microglia cell. The parenchyma also showed some immunostaining for the cleaved protein. Similar results were obtained in LPS-treated animals (not shown).

### Quantification of Neurons in the CA1 Str. Pyramidalis of Adult, Aged, aCSF- and LPS-Treated Rats

DAPI stained nuclei were counted in the ROI of CA1 Str. pyramidalis of adult, aged, aCSF- and LPS-treated rats. Results in Figure 10D show a significant decrease of the number of CA1 pyramidal neurons in aged rats as compared to the three other groups (−14%; One way ANOVA F_(3,35)_ = 9.265, P<0.0001; ***P<0.001 vs all other groups, Newman-Keuls multiple comparison test). Average thickness of CA1 Str. Pyramidalis was measured at three locations evenly distributed throughout the ROI of CA1 pyramidal cell layer and averaged values are reported in Figure 10E. We found that average thickness of CA1 pyramidal cell layer was significantly thinner (−15%) in aged rats as compared to all other groups (One way ANOVA F_(3,35)_ = 9.702, P<0.0001; ***P<0.01 vs all other groups, Newman-Keuls multiple comparison test). The average thickness of CA1 pyramidal cell layer was positively correlated with the average number of CA1 pyramidal neurons (Figure 10F, r^2^ = 0.6779; P<0.0001).

## Discussion

Aging is accompanied by a variety of neurobiological changes that contribute to a decline in cognitive function. Recently, the term "inflammaging" was coined [21] to characterize a widely accepted paradigm that ageing is accompanied by a low-grade chronic neuroinflammation. The hippocampus plays a critical role in memory formation and displays numerous electrophysiological, structural and morphological changes during normal and pathological ageing. In the present study, we characterized changes in the interactions between neurons, microglia and astrocytes within the CA1 region of the hippocampus during normal ageing processes and in response to experimental neuroinflammation induced by infusion of LPS, two conditions characterized by a chronic, low-grade inflammatory response.

Our results demonstrate that in the Str. Radiatum of aged rats GFAP protein levels were increased although astrocytes were less numerous and had distorted morphologies, with shorter and fragmented branches. Reactive and total numbers of microglia were decreased in aged rats while both increased in LPS-treated adult rats. In the CA1 and Str. Radiatum of aged rats many neurons showed signs of programmed cell death and were infiltrated by astrocyte branches which appeared to be actively bisecting the cell body into cellular debris. Neuronal debris was scattered throughout the Str. Radiatum of aged and adult LPS-treated rats. Reactive microglia within this region, possibly recruited by increased levels of the chemokine CX3CL1 [10], [22], [23], often contained neuronal debris within their cytoplasm, consistent with their scavenging activity of dying neurons. As a consequence, the number of CA1 pyramidal neurons was decreased in aged rats. Our results demonstrate that astrocytes and microglia in the hippocampus of aged and adult LPS-treated rats participate in the clearance of neuronal debris associated with programmed cell death and phagocytosis of apoptotic neurons.

GFAP protein levels increased in hippocampal homogenates of aged and LPS-treated rats. These results are in accordance with the literature which indicates that in the ageing brain, or in association with neurodegenerative diseases, trauma or ischemia, GFAP mRNA and GFAP protein expression increases [24]–[26]. The increased GFAP expression during ageing is due to increased transcription of GFAP [24], [25] which may be in response to oxidative stress [24], [27]. GFAP upregulation can occur either in the absence of proliferation or due to an increase in cell number [4]; we found that in the Str. Radiatum of aged rats increased GFAP expression was accompanied by a decreased number of astrocytes. Our possible explanation is that the observed differences between the levels of GFAP protein and the number of GFAP-positive astrocytes are due to differences in the proportion of soluble GFAP during aging [28], [29], rather than to differences in cell numbers per se since GFAP production in the aged hippocampus is mainly soluble GFAP and not the filamentous form [33]. Consistent with our results, previous studies reported no increase in the number of astrocytes in the hippocampus of male aged mice [30], [31] or male aged Brown Norway rats [32]. Furthermore, it has been reported that changes in GFAP transcription and expression [24] may alter the morphology of astrocytes and indirectly affect other cell types [33]. Our results also demonstrate that aged astrocytes had significantly shorter branches which appeared highly fragmented, a modification closely resembling the phenomenon of clasmatodendrosis described by Cajal [34]. In Alzheimer’s Disease and brain ischemia clasmatodendrosis may represent an acute astrocytic response to energy failure coupled to mitochondrial inhibition [20], [35], [36]. Given that differences in GFAP expression are indicative of differential functions of astrocytes with ageing [33], the aged rat astrocytes described herein, with their shrunken arborisation trees, may have lost their house-keeping functions of maintaining a scaffolding support and a viable environment for neighboring neurons may have acquired a role in helping clearing neuronal debris. Finally, in line with our results it has been shown that GFAP/vimentin KO results in an increase in cellular proliferation and neurogenesis in the granular layer of the dentate gyrus [37]. Furthermore, GFAP null astrocytes support neuronal survival and neurite outgrowth better than their wild-type counterparts [38], indicating that astrocyte senescence associated with increased GFAP expression can repress astrocytes’ capacity to increase neurogenesis and neuronal protection in the brain.

Several neurons in the aged and LPS-treated rat CA1 Str. Pyramidalis and CA1 Str. Radiatum appeared apoptotic, exhibiting modifications in proteins known to be involved in apoptosis. In accordance with the literature, we found significantly increased activation of p38MAPK [39] and increased immunostaining for CytC [40] and AIF [41] in the CA1 Str. Pyramidalis and CA1 Str. Radiatum of aged and LPS-treated rats. Some of the apoptotic neurons in the aged rats were surrounded and infiltrated by astrocyte branches, possibly recruited by Cx43 overexpression. Astrocyte branches were in close proximity to the apoptotic cells, embracing, infiltrating and wedging them to form debris. To the best of our knowledge this is the first demonstration that astrocyte branches become infiltrated within a neuron that shows signs of apoptosis, possibly for the purpose of bisecting the dying neuron and forming debris. Neuronal debris, scattered throughout the CA1 Str. Radiatum, were significantly more numerous in aged and LPS-treated rats than in adult rats and were all closely apposed to astrocyte branches. Overall, these results indicate that in normal brain ageing and, to a lesser extent, in LPS-induced inflammation in adult rats, apoptotic neurons are actively being phagocytized. These results are consistent with the hypothesis that both microglia and astrocytes can recognize danger signals in the surrounding parenchyma, and can help to clear apoptotic neuronal debris [42], [43]. Proper clearing of apoptotic cells and the resulting debris by phagocytizing glia may prevent injury to neighboring neurons [44], [45].

Reactive and total microglia decreased in the Str. radiatum of aged rats but increased in LPS-treated rats. Nevertheless, in aged rats we found several examples of microglia actively scavenging apoptotic neurons or containing phagocytised neuronal debris within their cytoplasm. Interestingly, CX3CL1 levels were increased in hippocampal homogenates of both aged and LPS-treated rats, and CX3CL1 immunostaining was present in neurons actively being phagocytized by microglia. CX3CL1 is expressed by neurons and is present in two different forms, with different functions: when embedded within the neuronal membranes CX3CL1 behaves as an adhesion molecule; in its cleaved and soluble form it is a chemoattractant recruiting CX3CR1-expressing microglia to injured neurons [9], [10], [22], [23] and regulating the phagocytic capacity of microglia [23], [46]–[48]. Signalling via CX3CL1 may underlie both the recruitment and activation of different glial cells and the well-organized topographic localization and spatial reciprocal interaction of microglia and astrocytes around apoptotic neurons. Although it has been shown that CX3CL1 contributes to the maintenance of microglia in a quiescent state (see [49], [50]) others reported that soluble CX3CL1 increases in cerebral ischemia [51], in response to apoptosis [52] and glutamate stimulation [22];it is neuroprotective in cultured rat hippocampal neurons [53] indicating that CX3CL1’s actions may differ depending upon different stimuli. Although it is still accepted that microglia play a critical role in establishing and maintaining inflammatory responses that may lead to neurodegenerative diseases [54] microglia also actively maintain their protective role during normal ageing [55]–[58] by clearing out dying cells [59]. Mouton and coworkers [30] found that astrocytes and CDllb-positive-microglia were increased in the CA1 of aged female mice in comparison to young female mice, but this effect was not observed in male mice [34]. The difference from our results may be due to species as well as sex differences.

We found a significantly higher number of total and reactive microglia in LPS-treated rats than in aged rats. We envisage that this phenomenon might mirror a defence reaction of the adult brain to the strong acute inflammatory stimulus in order to restore normal physiological conditions. Although the balance between reactive and normal microglia was different in aged with respect to LPS-treated adult rats, our results are consistent with the hypothesis that in both animal groups astrocytes and microglia were surveying the brain parenchyma possibly to prevent spreading of neuronal damage by apoptotic neurons and debris.

Impaired interplay between neurons and glia may be responsible for derangements from normal brain ageing to neurodegenerative processes [3], [4]. We discovered that in aged, but not in adult LPS-treated, rats the number of CA1 pyramidal neurons decreased significantly, and this decrease was highly correlated to thinning of the CA1 Str. Pyramidalis. During chronic low-grade neuroinflammation the loss of apoptotic neurons, accompanied by reduced neurogenesis in the subgranular zone of the dentate gyrus [60] may ultimately lead to the decreased number of CA1 neurons with ageing. This conclusion is supported by data demonstrating a decrease of astrocyte-dependent VEGF expression with ageing [61]. This effect may underlie the ageing-related decrease of cell plasticity that is normally maintained by the continuous addition of new neurons brought about by neurogenesis, and may contribute to senescence-dependent impairments of brain function. In LPS-treated adult rats we found that apoptotic neurons and debris were less numerous than in aged rats, indicating that either fewer CA1 neurons were undergoing apoptosis or that scavenging processes were more effective than in aged rats; this might be expected given the higher number of microglia. Longer time points of recovery after LPS treatment would be necessary to understand whether the abovementioned mechanisms would lead to recovery or neurodegeneration, since it has also been demonstrated that LPS is detrimental for neurogenesis in the adult rat brain [62].

We found that neurons which appeared to be infiltrated by astrocyte branches were located in the Str. Radiatum within 100 µm from CA1 Str. Pyramidalis which appeared indented in correspondence with the ectopic cell. We hypothesize that CA1 neurons that are in a late phase of the apoptotic process might be “removed” from the CA1 Str. pyramidalis, possibly by the astrocyte branches themselves via signalling with intercellular molecules such as the Cx43 and/or CX3CL1 [9]. This might represent a protective mechanism to avoid further spreading of toxicity and bystander damage of neighbouring pyramidal neurons in response to proinflammatory substances released into the parenchyma to facilitate microglia scavenging activity.

### Summary of Results Obtained

A summary of all the results obtained is reported in Table 1, in which the differences among the four experimental groups are reported. All the parameters found in the adult rat hippocampus were taken as controls and those found in aged, aCSF- and LPS-treated rats reported as percent of those found in adult rats.

### Conclusions

In conclusion, our results demonstrate that astrocytes and microglia in the hippocampus of aged and LPS-infused rats actively collaborate in the clearance of apoptotic neurons and neuronal debris associated with programmed cell death. The actions of astrocytes may represent either protective mechanisms to control inflammatory processes and the spread of further cellular damage to neighboring tissue or they may contribute to neuronal damage during pathological conditions. A better understanding of the benefits and risks of astrocyte and microglia activation is critical in order to determine whether future therapeutic interventions should attempt to enhance or impair the actions of glia.

## Supporting Information

Movie S1 **Movie shows the spatial rotation of the 3-D reconstruction of the confocal image shown in** **Figure 5D** **, taken in CA1 Str. Radiatum of an aged rat.** Immunoreactivity of GFAP (green) and NeuN (red) is shown.
(AVI)

Movie S2 **Movie shows the spatial rotation of the 3-D reconstruction of the confocal image shown in** **Figure 7B** **1–B2, taken in CA1 Str. Radiatum of an aged rat.** Immunoreactivity of GFAP (green) and CytC (red) is shown.
(AVI)

Movie S3 **Movie shows the spatial rotation of the 3-D reconstruction of the confocal image shown in** **Figure 8D** **4, taken in CA1 Str. Radiatum of an LPS-treated rat.** Immunoreactivity of GFAP (green), NeuN (red) and IBA1 (blue) is shown.
(AVI)

Movie S4 **Movie shows the spatial rotation of the 3-D reconstruction of the neuron-astrocyte-microglia “triad”, shown on the upper right quadrant of** **Figure 8D** **, taken in CA1 Str. Radiatum of an LPS-treated rat.** Immunoreactivity of GFAP (green), NeuN (red) and IBA1 (blue) is shown.
(AVI)

Movie S5 **Movie shows the spatial rotation of the 3-D reconstruction of a neuron-astrocyte-microglia “triad”, taken in CA1 Str. Radiatum of an LPS-treated rat.** Immunoreactivity of GFAP (green), NeuN (red) and IBA1 (blue) is shown.
(AVI)
